# Glycoengineering strategies for constructing defined Mucin O-glycans

**DOI:** 10.3389/fmolb.2026.1846568

**Published:** 2026-06-25

**Authors:** Thapakorn Jaroentomeechai, Matthew Phanchana, Kornchanok Jaiboon, Thanapong Kruangkum

**Affiliations:** 1 Department of Biotechnology, Faculty of Science, Mahidol University, Bangkok, Thailand; 2 Copenhagen Center for Glycocalyx Research, Danish National Research Foundation (DNRF196), University of Copenhagen, Copenhagen, Denmark; 3 Department of Molecular Tropical Medicine and Genetics, Faculty of Tropical Medicine, Mahidol University, Bangkok, Thailand; 4 Department of Anatomy, Faculty of Science, Mahidol University, Bangkok, Thailand; 5 Center of Excellence for Shrimp Molecular Biology and Biotechnology (CENTEX Shrimp), Faculty of Science, Mahidol University, Bangkok, Thailand

**Keywords:** chemoenzymatic synthesis, glycoengineering, glycopolymers, glycosyltransferases, mucin, O-glycosylation

## Abstract

Mucins are densely O-glycosylated proteins that establish mucosal barriers, mediate immune recognition, and serve as ligands for host lectins and microbes. However, mucin O-glycan structure-function relationships remain poorly defined due to inherent glycoform heterogeneity. Glycoengineering has emerged as a suite of methodologies enabling construction of mucins and mucin-type glycoconjugates with defined O-glycan structures suitable for rigorous biochemical and biophysical investigation. Here, we evaluate three glycoengineering approaches from a practical methodology perspective, emphasizing analytical validation requirements and method selection criteria. Cellular glycoengineering exploits genetic reconstruction of glycosylation pathways to produce simplified glycoforms in living cells, offering scalability and native protein context but limited homogeneity. Chemoenzymatic synthesis employs purified or cell-free-derived glycosyltransferases for sequential *in vitro* assembly with precise regiochemical and stereochemical control, though scalability remains constrained. Synthetic mucin mimetics utilize polymer chemistry to decouple glycan presentation from protein backbone constraints, enabling systematic variation of valency, density, and spacing. We discuss analytical validation workflows for each approach, and highlight emerging tools that may address current limitations in scalability and structural complexity. Together, we provide a decision framework that aligns experimental objective with the most appropriate glycoengineering platform.

## 1 Introduction

**FIGURE 1:**
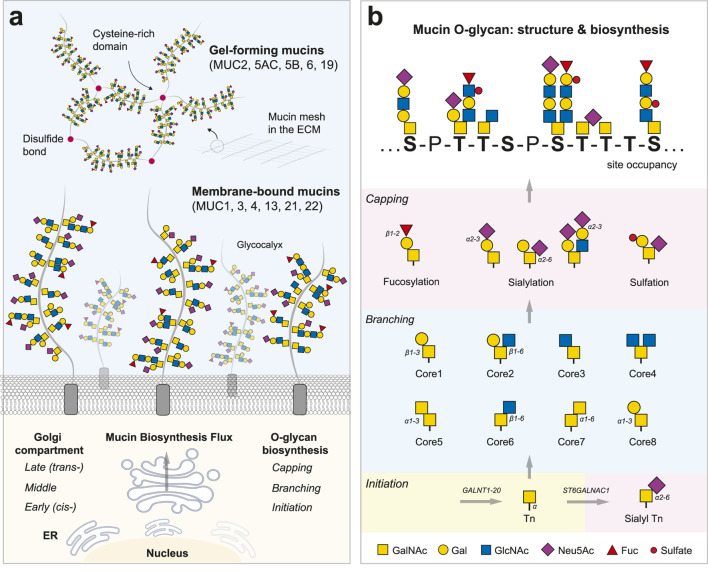
Biosynthesis of mucin and its O-glycans. **(a)** In humans, there are two major classes of mucins. Gel-forming mucins, including mucin-2 (MUC2), MUC5AC, MUC5B, MUC6, and MUC19, are secreted glycoproteins that oligomerize via disulfide bonds in cysteine-rich domains, forming a polymeric network that constitutes the mucus barrier as an important component in mucosal immunity. Membrane-bound mucins such as MUC1, MUC3, MUC4, MUC13, MUC21, and MUC22 are anchored to the cell surface and constitute the glycocalyx, where dense O-glycosylation generates a characteristic bottle-brush architecture. Mucin-type O-glycan biosynthesis proceeds through the Golgi compartments, with the initiation step occurring predominantly in the early (*cis*-) Golgi, core extension and branching in the middle Golgi, and capping in the late (*trans-*) Golgi network. Finally, mucins with mature O-glycans are then trafficked to the cell surface (membrane-bound) or secreted into the extracellular space (gel-forming). **(b)** Top - Structural heterogeneity of natural mucin O-glycans, arising from a vast sampling space in site-occupancy (macroheterogeneity) on the peptide backbone (S/T in bold as glycosylable sites) as well as monosaccharide compositions at a given site (microheterogeneity). Grey dashed boxes indicate sites of heterogeneity. Bottom - Schematic of mucin O-glycan biosynthesis. Initiation involves transfer of GalNAc to serine or threonine residues by polypeptide GalNAc-transferases (GALNT1-20), generating the GalNAc-α-O-S/T (Tn antigen) structure. Tn can be sialylated by ST6GALNAC1 enzyme to form sialyl-Tn. Extension proceeds through eight distinct core structures (Core 1–8) formed by specific glycosyltransferases (GTs). These core structures can be extended, and eventually capped with modifications such as fucosylation, sialylation, sulfation, and polyLacNAc formation. Representative human GT genes catalyzing the formation of each O-glycan structure are provided under its name. Specific GT genes catalyzing Core 5-8 formation remain ambiguous. Monosaccharide symbols follow the Symbol Nomenclature for Glycans (SNFG) (Varki et al., 2015).

Mucins are high-molecular-weight glycoproteins characterized by extensive O-glycosylation within tandem repeat (TR) domains enriched in proline (P), threonine (T), and serine (S) residues as a peptide backbone. This dense glycosylation modification on the TR domain, which can constitute up to 80% of the molecular mass, generates a bottle-brush architecture that constitutes a major component of the glycocalyx (Figure 1a) (Hansson, 2020; Luis and Hansson, 2023). Secreted gel-forming mucins establish the mucus barrier that lubricates epithelia and restricts pathogen access, while membrane-bound mucins constitute the apical glycocalyx that mediates cell signaling and immune surveillance (Hansson, 2020; Luis and Hansson, 2023; Corfield, 2015). Mucin O-glycans act as recognition motifs for endogenous lectins (e.g., Siglecs, galectins, macrophage galactose-type lectin or MGL) and as selective ligands for commensal and pathogenic microbes (Gustafsson and Hansson, 2025). Aberrant mucin O-glycosylation, exemplified by truncated Tn and sialyl-Tn antigens, is a hallmark of carcinomas (Stowell et al., 2015; Pinho and Reis, 2015) and is implicated in inflammatory bowel disease and cystic fibrosis (Luis and Hansson, 2023; Kang et al., 2022; Morrison et al., 2019). These observations underscore the importance of resolving structure-function relationships of mucin O-glycans. Yet, structural heterogeneity of mucin O-glycans, arising from combinatorial variation in monosaccharide composition, glycosidic linkage, chain length, branching pattern, and site occupancy, complicates their structure-function investigation (Figure 1b).

Mucin-type O-glycosylation initiates in the Golgi body with the transfer of *N*-acetylgalactosamine (GalNAc) to serine or threonine residues. In humans, this reaction is catalyzed by a family of 20 isoforms of polypeptide GalNAc-transferase (GalNAc-Ts) enzymes with overlapping substrate specificity (Schjoldager et al., 2020; Nielsen et al., 2022). Subsequent elongation by various glycosyltransferases generates distinct core structures, which can be further extended with polylactosamine chains and capped with terminal modifications including sialylation, fucosylation, and sulfation (Figures 1a, b) (Schjoldager et al., 2020). This so-called ‘non-template’ biosynthesis — driven by a multitude of factors including transferase enzyme expression levels, sugar substrate concentration, and spatial and temporal arrangement of Golgi architecture — yields extraordinary microheterogeneity where a single mucin species isolated from biological sources may comprise hundreds of distinct glycoforms, often reflecting differences in individual genetics, tissue origin, and physiological state (Bennett et al., 2012; Tran and Ten Hagen, 2013). The heterogeneity of natural mucins has limited mucin research to correlative observations rather than establishing structure-function relationships of mucin O-glycans. Thus, one of the current challenges in the field is the availability of mucin glycoconjugates with defined O-glycan structures, in particular, a control over glycan regiochemistry, anomeric stereochemistry, chain elongation, branching, and spatial presentation on the polypeptide or polymer backbone. Glycoengineering strategies that can deliver homogeneous or structurally defined materials are therefore essential for biophysical characterization, binding studies, and functional assays.

In the past few decades, three glycoengineering approaches have emerged: (a) cellular glycoengineering, which leverages precise genetic manipulation of living cells to simplify and reconstruct glycosylation pathways; (b) chemoenzymatic synthesis, which employs purified or cell-free-produced glycosyltransferases for stepwise *in vitro* glycan assembly; and (c) synthetic mucin mimetics, which utilize chemical synthesis to generate polymer scaffolds with defined glycan structure and presentation. Each approach offers distinct advantages in terms of chemical control, scalability, and biological relevance. This work provides an overview of recent progress in each strategy and evaluates each approach from a practical standpoint, emphasizing analytical validation requirements and providing decision criteria for method selection.

## 2 Analytical prerequisites for mucin characterization

Comprehensive characterization of both natural and synthetic mucins requires specialized analytical platforms capable of resolving the structural complexity inherent to these glycoconjugates. Recent technological advancements have substantially improved our ability to dissect and visualize mucin microheterogeneity. Mucin-selective proteases (mucinases) such as the StcE and SmE have enabled site-specific glycopeptide mass spectrometry (MS) analysis by cleaving within densely glycosylated domains that resist conventional protease digestion (Chongsaritsinsuk et al., 2023; Malaker et al., 2019). MS-based O-glycoproteomics workflows that combine mucinase digestion with electron-based fragmentation now permit confident identification of O-glycosites (Mahoney and Malaker, 2024), while ion mobility-tandem MS provides a high-throughput platform for separating and characterizing O-glycan isomers from complex biological samples (Bechtella et al., 2024). Other emerging techniques including single-molecule imaging have enabled direct visualization of intact glycans on glycoproteins and glycolipids, providing monosaccharide-level structural resolution on individual mucin molecule (Anggara et al., 2023; Wu et al., 2020). These analytical advances are prerequisites for validating synthetic, and eventually natural, constructs and establishing structure-function relationships of mucin O-glycans. For comprehensive reviews of mucin analytical methods, readers are referred to recent reviews on glycoproteomics (Bagdonaite et al., 2022) and mucinomics (Rangel-Angarita and Malaker, 2021).

The choice of analytical platform should be matched to the glycoengineering strategy. Cell-derived mucin glycoforms require glycomics and glycoproteomics in combination with cell-population profiling (flow cytometry, lectin staining, and increasingly single-cell glycomics) to verify both glycan structure and population homogeneity. Chemoenzymatically synthesized glycopeptides are characterized by site-specific MS and nuclear magnetic resonance (NMR) spectroscopy analysis for atomic-level confirmation of regio- and stereochemistry at each glycosidic linkage. Mimetic glycopolymers require size-exclusion chromatography with multi-angle light scattering (SEC-MALS), NMR, and lectin profiling to establish the degree of polymerization, glycan loading, and bioactivity. These analytical prerequisites are summarized alongside platform-specific considerations in the discussion section.

## 3 Cellular glycoengineering: hijacking biosynthetic machinery

Cellular glycoengineering exploits the endogenous glycosylation machinery of living cells through targeted genetic manipulation. This approach involves deletion of competing biosynthetic pathways to channel metabolic flux toward desired glycan structures, thereby simplifying the glycoform output while preserving native protein folding, trafficking, and other post-translational processing such as disulfide bond formation. The advent of precise genetic engineering, in particular zinc-finger nucleases (ZFNs) and clustered regularly interspaced short palindromic repeats (CRISPR) systems along with established glycoengineering resources, has now made cellular glycoengineering accessible to a wider community (Clausen et al., 2022; Huang H. W. et al., 2025; Kao et al., 2024).

**FIGURE 2:**
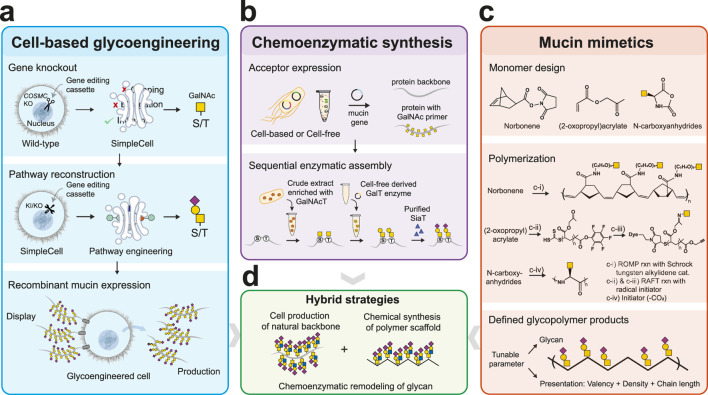
Three glycoengineering strategies for constructing defined mucin-type O-glycans. **(a)** Cell-based glycoengineering employs CRISPR-Cas9-mediated knock-out (KO) of the COSMC gene to generate SimpleCell lines that produce only truncated Tn antigen (GalNAc-α-O-Ser/Thr), eliminating downstream core extension and capping. Sequential knock-in (KI) or reconstitution of specific glycosyltransferase genes (e.g., *C1GALT1, GCNT1*, *ST3GAL1*) enables stepwise pathway reconstruction to produce defined core structures with controlled elongation and terminal modifications. Glycoengineered cells can display recombinant mucins on the cell surface for binding assays or secrete them for biochemical characterization. Note that prokaryotic cellular glycoengineering for mucin O-glycan production follows distinct principles (cytoplasmic GalNAc-T2/Gne pathway or O-OST/LLO transfer) and is summarized in Table 1 and Section 3. **(b)** Chemoenzymatic synthesis utilizes cell-based or cell-free expression systems to produce glycosyltransferases (e.g., polypeptide *N*-acetylgalactosaminyltransferase (ppGalNAcT)) and acceptor substrates bearing mucin peptide backbones with Tn initiation. Sequential *in vitro* assembly employs crude cell extracts enriched with specific transferases, cell-free-derived enzymes, or purified glycosyltransferases such as galactosyltransferase (GalT), or sialyltransferase (SiaT) together with nucleotide-sugar donors to achieve stepwise glycan elaboration with defined regio- and stereo-chemistry at each glycosidic linkage. **(c)** Synthetic mucin mimetics employ polymer chemistry to decouple glycan presentation from native protein backbone constraints. Representative glycan-functionalized monomers including norbornene derivatives, (2-oxopropyl)acrylates, and N-carboxyanhydrides undergo controlled polymerization via: c-i) ring-opening metathesis polymerization (ROMP) with ruthenium-based Grubbs catalysts or molybdenum/tungsten Schrock alkylidenes for stereo-controlled polymerization; c-ii), c-iii) reversible addition-fragmentation chain transfer (RAFT) polymerization with radical initiators; or c-iv) N-carboxyanhydride (NCA) ring-opening polymerization, yielding glycopolymers with tunable valency, density, and chain length. **(d)** Hybrid strategies combine either cell-based production of natural mucin backbones or chemical synthesis to make mucin mimetic scaffolds, and then chemoenzymatic remodeling of pendant glycans, to generate materials that integrate the structural authenticity of enzymatic glycosylation with the tunability of mucin mimetic platforms. Monosaccharide symbols follow the Symbol Nomenclature for Glycans (SNFG).

In mammalian systems, the SimpleCell strategy exemplifies this approach. This strategy leverages CRISPR/Cas9 system to knock out (KO) the *COSMC* gene. *COSMC* encodes the chaperone required for Core 1 synthase (C1GALT1) activity, and its KO thereby produces cell lines that exclusively generate truncated Tn antigen as O-glycan (Figures 1b, 2a) (Steentoft et al., 2011). This single genetic perturbation eliminates downstream core extensions and capping, yielding O-glycoproteins bearing only the initiating GalNAc residue. Cells with simplified, homogeneous Tn O-glycan have permitted systematic mapping of O-glycosites within the proteomes of human and other organisms (Steentoft et al., 2013; Yang et al., 2014; Bagdonaite et al., 2015).

Sequential knock-in/knock-out (KI/KO) of specific glycosyltransferase (GT) genes extends the SimpleCell concept to construct isogenic cell line libraries producing defined O-glycan structures with controlled elongation and capping glycans (Table 1) (Konstantinidi et al., 2022; Narimatsu et al., 2019; Nason et al., 2021). For example, KI of *Hs*B3GNT6 in the SimpleCell background installs Core 3 O-glycan, while KI of *Hs*GCNT1 in wild-type CHO cells reconstructs Core 2 O-glycan and its sialylated forms (Nason et al., 2021; Jaroentomeechai et al., 2024). Beyond core structure, the same engineering principle has been recently extended to construct cells capable of modifying the non-reducing end of O-glycans with sialylation, fucosylation, and sulfation. These terminal modifications often define epitopes for lectins, selectins, Siglecs, and microbial adhesins (Schjoldager et al., 2020). For example, the introduction of human FUT2 enzyme into glycoengineered HEK293 cells enabled cell-based production of Type 3 H histo-blood group structures (Fucα1-2Galβ1-3GalNAcα) on mucin-like reporters (Jaroentomeechai et al., 2024). Similarly, Sun *et al* demonstrated that expression of human sulfotransferases (CHST1/3, GAL3ST2/4) in HEK293 SimpleCell background allows for a display and production of several human mucins bearing 3-*O*- or 6-*O*-sulfoepitopes (Sun et al., 2022).

**TABLE 1:** Representative glycosyltransferases and sulfotransferases employed in mucin O-glycoengineering.

Name^a^	Key enzymes^b^	Glycoengineering strategy
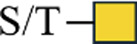 **Tn** (GalNAcα-O-Ser/Thr)	*Hs*ppGalNAc-Ts	Cellular KO: *COSMC* in mammalian cells and co-expression with human mucin or O-Glycocarrier reporters (Konstantinidi et al., 2022; Nason et al., 2021; Jaroentomeechai et al., 2024) Overexpression of *Hs*ppGalNAcT2 in *E. coli* (Du et al., 2019; Wardman et al., 2023) Chemoenzymatic Purified ppGalNAcT combined with UDP-GalNAc and mucin peptide substrate (e.g., (Sorensen et al., 2006; Tarp et al., 2007), for review, see (Sanz-Martinez et al., 2023))
	O-OST system (O-glycosylation: *Nm* and *Ng*OST, LLOs assembly: *Ab*PglC)	Leveraging bacterial O-OST for an *en bloc* transfer of O-glycans in *E. coli*, requiring preassembled glycan on lipid to create lipid-linked oligosaccharides substrate (LLOs) (Natarajan et al., 2020)
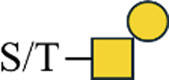 **Core 1 (T)** (Galβ1-3GalNAcα-O-Ser/Thr)	*Hs*C1GalT, *Hs*Cosmc, *Dm*C1GalT1	Cellular Mammalian cells with KO: *GCNT1*, *ST6GALNACs*, and *ST3GALs* and co-expression with human mucin or O-Glycocarrier reporters (Nason et al., 2021; Jaroentomeechai et al., 2024) Overexpression of *Hs*ppGalNAcT2, *Cj*CgtB, and *Cj*Gne in *E. coli* (Du et al., 2019) Chemoenzymatic Use recombinant *Dm*C1GalT with UDP-Gal to modify Tn-glycopeptide acceptor (Gonzalez-Ramirez et al., 2022; Wang et al., 2021)
	O-OST system (LLOs assembly: Tn biosynthesis pathway plus *Ec*WbwC)	Cellular & Chemoenzymatic Co-expression of *Ec*WbwC generated core1-LLOs that can be transferred by O-OST. The similar reaction has been demonstrated in cell-free system (Natarajan et al., 2020)
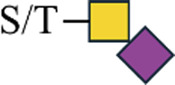 **STn** (Neu5Acα2-6GalNAcα-O-Ser/Thr)	*Hs*ST6GalNAc1-2, *Pd*2,6ST	Cellular Overexpression of *Hs*ST6GalNAc1 in human cell lines to produce MUC1-STn on cell surface (Marcos et al., 2011) Reconstitution of *Hs*ST6GalNAc1 in HEK293 KO: *COSMC* (SimpleCell) backgrounds and co-expression with human mucin or O-Glycocarrier reporters (Nason et al., 2021; Jaroentomeechai et al., 2024) Chemoenzymatic OPME assembly using bacterial *Pd*2,6ST with CMP-sialic acid synthetase (*Nm*CSS) and sialic acid aldolase (*Ec*NanA) to sialylate MUC1-Tn glycopeptide (Wang et al., 2021; Malekan et al., 2013) Use recombinant *Hs*ST6GalNAc1 to install STn with biotin handle on a surface of the living cell (Yang et al., 2023)
	O-OST system (LLOs assembly: Tn biosynthesis pathway plus *Ec*WbwA, *Psp*2,6ST)	Cellular Co-expression of bacterial α2,6-sialyltransferases generated STn-LLOs that can be transferred by O-OST, albeit at low efficiency (Natarajan et al., 2020)
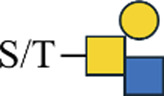 **Core 2** (GlcNAcβ1-6 [Galβ1-3]GalNAcα-O-Ser/Thr)	*Hs*GCNT1/3/4	Cellular Certain human lines (e.g., HEK293) naturally produce core 2 O-glycan which can be installed on mucin reporters following co-expression (Nason et al., 2021) CHO cell with KI: *Hs*GCNT1 and co-expression with O-Glycocarrier reporter (Jaroentomeechai et al., 2024) Chemoenzymatic Recombinant GCNT1 on T-antigen acceptor with UDP-GlcNAc, one-pot assembly using OPME cascades (Ma W. et al., 2022; Xu et al., 2021; Santra et al., 2019)
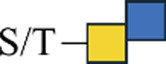 **Core 3** (GlcNAcβ1-3GalNAcα-O-Ser/Thr)	*Hs*B3GNT6 (Core 3 synthase)	Cellular Overexpression of *Hs*B3GNT6 in human cell line with KO: *COSMC* or KO: *C1GALT1* and co-expression with human mucin or O-Glycocarrier reporters (Nason et al., 2021; Jaroentomeechai et al., 2024) Chemical synthesis Total chemical synthesis of core 3 and chemoenzymatic elaborations to other structures using CEMA (Wang et al., 2021)
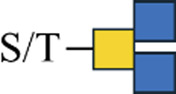 **Core 4** (GlcNAcβ1-6 [GlcNAcβ1-3]GalNAcα-O-Ser/Thr)	*Hs*GCNT3 (bifunctional Core 2/Core 4 synthase)	Cellular Overexpression of *Hs*GCNT1 and *Hs*B3GNT6 in CHO cell to produce PSGL-1 with higher proportion of core 4 modification (Cherian et al., 2015). Note that conventional Core 4 synthase (GCNT3) was not used in this study Chemical synthesis Total chemical synthesis of core 3 and chemoenzymatic elaborations to other structures using CEMA (Wang et al., 2021)
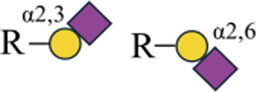 **Sialylation** (α2,3/α2,6-Neu5Ac capping) e.g., sialyl-T, sialyl-LacNAc	α2,3 linkage: *Hs*ST3Gal1, *Hs*ST3Gal4 *Pm*ST1, *Pm*ST3, *Cj*Cst-I α2,6 linkage: *Hs*ST6Gal1, *Pd*2,6ST, *Psp*2,6ST	Cellular Combinatorial KI/KO of *Hs*ST3Gal/*Hs*ST6Gal isoforms in HEK293 expressing mucin panels (Bull et al., 2021) Combinatorial KI/KO. For example, KO of *St6galnac4* gene in CHO cells to generate sialyl core 1 structure and co-expression with O-Glycocarrier reporters (Jaroentomeechai et al., 2024) Overexpression of *Hs*ST3Gal1 or *Cj*CsT-I in glycoengineered *E. coli* (Sim et al., 2022) Chemoenzymatic OPME systems combining sialic acid aldolase, CMP-sialic acid synthetase (*Nm*CSS), and a sialyltransferase to install α2,3- or α2,6-Neu5Ac (and Neu5Gc, Kdn, non-natural variants) (Chen, 2024) CEMA strategy using *Pm*ST1, *Pd*2,6ST, *Hs*ST6GalNAc4 on synthetic core substrates (Wang et al., 2021)
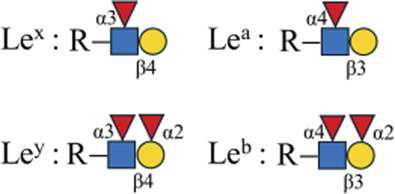 **Fucosylation** (α1,2/α1,3/α1,4-Fuc capping) e.g., Lewis X, Lewis A, sialyl-Lewis X	α1,2-fucosyltransferases: *Hs*FUT1, *Hs*FUT2, *Hm*2FucT α1,3/α1,4-fucosyltransferases: *Hs*FUT3, FUT5, FUT6, FUT7, *Hp*FucT	Cellular Knock-in of *Hs*FUT2 isoforms in glycoengineered HEK293 to install Lewis-type epitopes on mucin O-glycans (Jaroentomeechai et al., 2024) Chemoenzymatic Recombinant *Hs*-, *Hm*-, or *Hp*-FUTs on type-1 (Galβ1-3GlcNAc) or type-2 (Galβ1-4GlcNAc) acceptors with GDP-Fuc, divergent assembly of Lewis repertoires (Wang et al., 2021; Bai et al., 2025a)
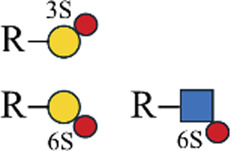 **Sulfation** (6-O-sulfo-GlcNAc, 3-/6-O-sulfo-Gal)	GlcNAc-6-*O*-sulfotransferases: *Hs*CHST2, *Hs*CHST4 Gal-6-*O*-sulfotransferase: *Hs*CHST1 Gal-3-*O*-sulfotransferase: *Hs*Gal3ST1/2	Cellular Expression of *Hs*CHST1, *Hs*CHST2, or *Hs*Gal3ST2 in wild-type or glycoengineered cells to produce or display mucins with sulfation for immune receptor functional studies (Sun et al., 2022; Goerdeler et al., 2026; Jung et al., 2025) Chemoenzymatic Recombinant *Hs*CHST2 with PAPS donor on human CD34 O-glycopeptide (Jiang et al., 2026)

^a^ Core 5-8 are rare cores with incomplete biosynthetic enzyme identification. Therefore, we did not include these cores in the table. However, total chemical synthesis of these cores was recently reported (Wang et al., 2021).

^b^ Abbreviation: *Hs, Homo sapiens; Dm, Drosophila melanogaster; Pd, Photobacterium damselae; Psp, Photobacterium* spp.*; Pm, Pasteurella multocida; Cj, Campylobacter jejuni; Hm, Helicobacter mustelae; Hp, Helicobacter pylori; Nm, Neisseria meningitidis; Ng, Neisseria gonorrhoeae; Ec, Escherichia coli; Ab, Acinetobacter baumannii.* S, serine; T, threonine; R, any glycan moieties; KO, knockout; KI, knock-in; OPME, one-pot multienzyme; CEMA, chemoenzymatic modular assembly; LLO, lipid-linked oligosaccharide; O-OST, O-linked oligosaccharyltransferase; PAPS, 3′-phosphoadenosine-5′-phosphosulfate; SimpleCell, COSMC-knockout cell line displaying truncated Tn antigen.

These glycoengineered cell lines serve as either a display platform for cell-based glycan array studies (Narimatsu et al., 2019) or a production platform for recombinant human mucins and mucin-like domains (Konstantinidi et al., 2022; Nason et al., 2021), as well as synthetic glycodomains (Jaroentomeechai et al., 2024), bearing defined O-glycan structures. Structurally-defined mucin materials are essential for high-resolution characterization of mucin (Anggara et al., 2023; Kearns et al., 2024) and for dissecting glycan-dependent recognition of mucins by immune receptors such as Siglecs (Bull et al., 2021; Goerdeler et al., 2026) and MGL (Grosso et al., 2025), by mucin-degrading enzymes (Narimatsu et al., 2024; Narimatsu et al., 2026), as well as by microbial toxins and adhesins (Elzinga et al., 2024; Chen et al., 2025; Jaroentomeechai et al., 2025). Further advancement in genetic and cellular engineering including multiplexed CRISPR-based KI/KO strategies, and inducible genetic circuits enabling tunable regulation of glycosylation pathways (Lim et al., 2025), combined with deeper understanding of spatial control of glycosyltransferase localization within the Golgi compartments (Yagi et al., 2024; Harada et al., 2024), and of substrate specificities of GalNAc-T isoform toward complex mucin acceptors (Coelho et al., 2022), will accelerate the development of glycoengineered cell libraries that are readily deployable for producing mucins with more complex O-glycan structures that can faithfully reflect their natural glycan epitopes.

Beyond mammalian cells, prokaryotic systems offer advantages in terms of genetic tractability and scalability, and certain bacterial systems have been engineered to produce defined mucin-type O-glycans (Palma et al., 2024; Natarajan et al., 2018). Most prokaryotes including laboratory *Escherichia coli* strains lack endogenous protein glycosylation pathway, providing a blank canvas for glycosylation pathway construction. While much progress has been made in engineering *E. coli* for N-glycan and N-glycoconjugate production (Palma et al., 2024) including the biosynthesis of authentic human Man_3_GlcNAc_2_ N-glycan core structure (Valderrama-Rincon et al., 2012) and its installation at authentic human N-glycosites (Ollis et al., 2015), reports using a similar strategy to generate glycoengineered *E. coli* strains capable of O-glycan production are sparse. To date, two mechanistically distinct strategies have been implemented for bacterial O-glycoprotein production. The first involves direct co-expression of human polypeptide N-acetylgalactosaminyltransferase 2 (GalNAc-T2) with a UDP-GlcNAc/GalNAc 4-epimerase (e.g., Gne from *Campylobacter jejuni*) that converts endogenous UDP-GlcNAc to the required donor UDP-GalNAc (Bernatchez et al., 2005). This enables cytoplasmic installation of GalNAcα1-O-Ser/Thr (Tn antigen) on recombinant substrates (Du et al., 2019). Sequential co-expression of elongating and capping enzymes, including bacterial β1,3-galactosyltransferase CgtB from *C. jejuni* (Bernatchez et al., 2007) and mammalian sialyltransferases (e.g., ST3Gal1, ST6GalNAc2, ST6GalNAc4), permits further elaboration to T, sialyl-T, and di-sialyl-T glycoforms on acceptors including human interferon α2b and human growth hormone (Du et al., 2019; Sim et al., 2022). Recently, glycoengineered *E. coli* has been deployed as a FRET-based screening platform to functionally assay and evolve O-glycopeptidases (Wardman et al., 2023).

The second strategy employs a bacterial oligosaccharyltransferase (O-OST) from *Neisseria* species as catalyst for the O-glycosylation pathway. O-OST requires a pre-assembled lipid-linked oligosaccharide (LLO) as the donor substrate (Faridmoayer et al., 2008). PglC from *Acinetobacter baumannii* can be used to assemble GalNAc onto undecaprenyl pyrophosphate (Und-PP) to generate Tn-LLOs, which will then be flipped to the periplasmic space by *E. coli* Wzx flippase, and then *en bloc* transferred to acceptor sequences by O-OST (Natarajan et al., 2020). Co-expression of extension enzyme such as *E. coli* WbwC generates Core1-LLOs which can be utilized by O-OST. This OST-based route enabled the production of antigenically authentic Tn-MUC1 glycoforms as confirmed by reactivity with the cancer-specific anti-Tn-MUC1 antibody 5E5 (Sorensen et al., 2006). Notably, O-OSTs are found to be highly promiscuous (Faridmoayer et al., 2008) and it is anticipated that a large repertoire of O-glycan structures can be transferred by O-OSTs. Overall, cellular glycoengineering in prokaryotic platform offers advantages in scalability, genetic tractability, and the absence of competing endogenous pathways, though bottlenecks including protein folding, disulfide bond formation, and the availability of nucleotide sugar donors may require further strain engineering and optimization to overcome (Natarajan et al., 2018; Kay et al., 2022).

The chemical control achievable through cellular glycoengineering includes glycan truncation, core structure selection, and modulation of site occupancy by GalNAcT isoform expression. Control over terminal modifications (sialylation, fucosylation, and sulfation) is achieved through knockout or overexpression of the relevant transferases (Sun et al., 2022; Goerdeler et al., 2026). Owing to their scalability, glycoengineered cells can be leveraged for high-throughput screening to discover and improve glycosylation-related enzymes and regulators in both mammalian (Tsui et al., 2024) and prokaryotic systems (Wardman et al., 2023; Glasscock et al., 2018). However, limitations persist. Key challenges include achieving complete glycoform homogeneity and expanding access to non-natural glycan modifications. Homogeneity is limited due to residual glycosyltransferase activities, variable site occupancy, and cell-to-cell variation within populations. Non-natural sugar substrates exhibit variable membrane permeability and metabolic incorporation efficiency, limiting access to modified glycan structures (Bull et al., 2017). Additionally, accessible glycan structures are constrained by the substrate specificities of available glycosyltransferases and the metabolic capacity of the host cell. Future directions include development of improved genetic circuits for the coordinated expression of glycosylation machinery, engineering of glycoenzymes with altered selectivity and improved activity (Schumann et al., 2020), and metabolic engineering to enhance nucleotide sugar donor availability (Natarajan et al., 2018). Combining CRISPR-based screening with glycoproteomics may enable systematic identification of genetic determinants governing glycosylation (Romer et al., 2023).

## 4 Chemoenzymatic synthesis: precision assembly *in vitro*

Chemoenzymatic synthesis assembles defined glycans through stepwise enzymatic reactions on synthetic or semi-synthetic acceptors *in vitro*. The required GTs can be supplied from conventional recombinant expression, cell-free protein synthesis (CFPS), or crude lysates enriched with glycosylation machinery (Overkleeft et al., 2022; Jaroentomeechai et al., 2020). Compared to cellular approaches, this strategy decouples glycan assembly from cellular metabolism, enabling defined regiochemistry and stereochemistry at each glycosidic linkage and direct access to non-natural sugar donors and acceptors.

The modular assembly strategy involves sequential enzymatic transformations starting from GalNAcα-O-Ser/Thr acceptors, which may be prepared by chemical synthesis or enzymatic glycosylation of synthetic peptides (Sanz-Martinez et al., 2023). Subsequent elongation and branching to generate other core structures are achieved through employing appropriate transferases, for example, *Dm*C1GalT1 for Core 1, and *Hs*GCNT1 for Core 2 branching, together with cognate nucleotide-sugar donors (Table 1; Figure 2b) (Wang et al., 2021; Gadi et al., 2023). Wang and colleagues recently established a chemoenzymatic modular assembly (CEMA) framework in which a small set of common precursors (Core 1–8) is divergently elaborated by GT cascades to access extended Core 1–4 (Wang et al., 2021) and the rare Cores 5, 7, and 8 (Gadi et al., 2023). Terminal modifications of core O-glycans including α2,3- and α2,6-sialylation by ST3Gal and ST6GalNAc enzyme family, α1,3/4-fucosylation by FUT3/4 enzymes, and 6-O-sulfation by CHST enzymes (Table 1) generate glycan epitopes suitable for functional studies such as Siglec interaction (Wang et al., 2021; Gadi et al., 2023; Xu et al., 2024). For mucin-specific glycopeptides, this strategy was used to produce MUC1 tandem repeat fragments bearing Tn, T, sialyl-T, and STn antigens for cancer vaccine evaluation (Sorensen et al., 2006; Tarp et al., 2007; Teng et al., 2025) and for cell adhesion studies (Bello et al., 2023). As a notable application of this strategy, highly O-glycosylated MUC7 glycopeptides bearing glycans (Tn, T, sialyl-T, and fucosylated-T) at all nine glycosylation sites were chemoenzymatically synthesized to reveal sialic-acid-dependent inhibition of *Pseudomonas aeruginosa* biofilm formation (Ma et al., 2025), illustrating how precise glycoform control enables direct mechanistic dissection of glycan-dependent function.

One-pot multienzyme (OPME) cascades have been developed where sequential transformations proceed without intermediate purification, improving synthetic efficiency and reducing handling losses (Sperl and Sieber, 2018). Careful optimization of enzyme ratios, donor concentrations, and reaction conditions enables near-quantitative conversion through multiple sequential glycosylation steps. Such cascades have recently been implemented to generate repertoires of human milk oligosaccharides (Bai Y. et al., 2025), and enable on-demand regeneration of nucleotide sugars from inexpensive starting materials (Elling, 2025; Hoang et al., 2025). Recently, Liu *et al* developed the stop-and-go strategy employing unnatural sugar donors with MGAT4/5 to produce complex N-glycans (Liu et al., 2019). Although developed for asymmetric N-glycan branching, their principle of using kinetically distinct donors for regiocontrol may be transferable to mucin O-glycan branching.

Central to scaling chemoenzymatic approaches is the access to diverse GTs. Large-scale efforts have been described for recombinant expression of GTs in yeast, insect, and human cell lines (Shimma et al., 2006; Moremen et al., 2018). However, conventional recombinant expression of mammalian GTs is often hampered by misfolding, poor solubility, and post-translational modification requirements that exceed bacterial capacity. *E. coli* cell-free protein synthesis (CFPS) circumvents these limitations by performing transcription–translation in an open environment that can be supplemented with chaperones, redox buffers, and substrates. Moreover, the ability to obtain large repertoire of useful GTs from a simple prokaryotic culture will accelerate functional studies and utilization of GTs. Two distinct *E. coli*-based cell-free strategies have been developed to address this need. *E. coli*-based CFPS systems enable rapid, on-demand expression of individual GTs including those that are otherwise difficult to obtain by conventional recombinant expression. Further, protein engineering strategies such as fusion partners (Jaroentomeechai et al., 2022; Kwon et al., 2026) or computational design (Go et al., 2023) can be deployed to enhance soluble expression of diverse GTs directly in the *E. coli* CFPS reaction. Alternatively, crude *E. coli* lysates enriched with glycosylation machinery couple protein synthesis and glycosylation within a single reaction vessel, enabling rapid prototyping of glycosylation pathways without enzyme purification (Schoborg et al., 2018; Stark et al., 2023; Jaroentomeechai et al., 2018; Kightlinger et al., 2019). The open reaction environment permits supplementation with cofactors, metal ions, and nucleotide-sugar donors at optimized concentrations.

Chemoenzymatic synthesis provides a high degree of structural control among current glycoengineering approaches. Site-specific glycosylation on synthetic peptide scaffolds is readily achieved by controlling acceptor peptide sequence and GalNAc-T isoform selection, enabling construction of glycopeptide libraries with systematic variation in glycan structure, attachment site, and glycan density. Such materials have enabled X-ray crystallographic and cryo-EM structural studies of glycan-protein interactions previously inaccessible due to sample heterogeneity (Gonzalez-Ramirez et al., 2022; Bello et al., 2023). The high-resolution structural information obtained from these studies has revealed molecular details of lectin-glycan recognition, including the contribution of the peptide backbone to binding affinity and specificity (Sanz-Martinez et al., 2023; Ma et al., 2025).

Certain limitations persist in scaling up chemoenzymatic glycan synthesis, in particular the cost of nucleotide sugar substrates and the labor-intensive nature of stepwise synthesis. These are being addressed by emerging technologies that span cell-free, computational, and automation domains. Enzymatic regeneration cascades for UDP-GalNAc (Hoang et al., 2025) and broader nucleotide-sugar systems (Elling, 2025) have reduced donor costs by leveraging inexpensive starting materials such as sucrose. Automated chemoenzymatic platforms now interface enzymatic glycan elaboration with solid-phase or microfluidic infrastructure (Overkleeft et al., 2022; Suri and Gilmour, 2025; Guberman and Seeberger, 2019). Using digital microfluidic OPME synthesis, Wu *et al* reported production of human milk oligosaccharides (HMOs) with highest yields exceeding 90% and with significant reduction in time and labor (Wu et al., 2024). Synthesis efficiency can also be improved using machine learning-guided enzyme engineering. Landwehr *et al* integrated cell-free DNA assembly, CFPS, and high-throughput functional assays to map sequence-fitness landscapes for amide synthetases and predict variants with up to 42-fold improved activity, providing a platform for evolving other enzyme classes including GT families (Lan et al., 2025). Machine learning has also been used to predict substrate specificity for GT1 family using UDP-glucose (Harding-Larsen et al., 2024), and similar approaches are anticipated for mucin GT families such as GT27 (Drula et al., 2022). Together, these advances outline a plausible path toward gram-scale, automated chemoenzymatic synthesis of defined mucin glycopeptides with well-defined O-glycans.

## 5 Synthetic mucin mimetics: polymer chemistry approaches

Mucin mimetics substitute the native polypeptide backbone with synthetic polymer scaffolds, decoupling glycan presentation from protein sequence constraints. This approach enables systematic investigation of how physical parameters including valency, glycan density, inter-glycan spacing, and backbone rigidity influence biological function independent of peptide sequence-specific effects (Kohout et al., 2022). In 2010, Godula and Bertozzi reported synthetic strategy for glycopolymer using reversible addition-fragmentation chain transfer (RAFT) polymerization of acetoxime acrylate monomers with biotinylated chain transfer reagent to produce poly (acryloyl hydrazide) scaffolds bearing chain-end functional groups, which allows for site specific ligation onto microarray. Reducing glycans were then conjugated along polymer scaffold via hydrazone chemistry (Godula and Bertozzi, 2010). Subsequent work from the same group introduced a chemically synthesized mucin domain with preserved native glycosidic bonds (Kramer et al., 2015). The synthetic mucin can be modified with chemical handles at both N- and C-terminals, allowing site-specific docking of synthetic mucin domain on the surface of the living cells (Kramer et al., 2015). Notably, equipping synthetic mucin domain with phospholipid permits direct remodeling of glycocalyx on cell surface, and this strategy was used to understand how degree of sialylation on cell surface can modulate Siglec interaction, and thus the immunosuppressive properties of the carcinoma cells (Hudak et al., 2014). These studies established that synthetic mucin moieties, even with non-native or hybrid backbones, can recapitulate mucin-dependent biological activity.

Typically, polymerization methods determine backbone architecture, while post-polymerization conjugation chemistries graft glycans onto pre-formed scaffolds. Among polymerization methods, ring-opening metathesis polymerization (ROMP) is used to generate a rigid polynorbornene backbone that enforces extended conformations, mimicking the bottle-brush architecture of native mucins (Kruger et al., 2021). Norbornene-based monomers bearing protected glycan moieties undergo living polymerization with ruthenium or molybdenum/tungsten carbene catalysts, yielding polymers with controlled chain length and dispersity through monomer-to-catalyst stoichiometry (Figure 2c) (Kruger et al., 2021). Controlled radical polymerization techniques, including atom transfer radical polymerization (ATRP) and RAFT polymerization, provide complementary access to branched, star, and brush architectures (Kohout et al., 2022; Godula and Bertozzi, 2010). Alternatively, N-carboxyanhydride ring-opening polymerization (NCA-ROP) offers a synthetic route that yields polymers featuring natural peptide linkages (Kramer and Deming, 2010). This strategy has been deployed to generate mucins with regio- and stereo-specific presentation of O-glycan (Wood et al., 2025) as well as mucin with unnatural stereoisomers (Kohout et al., 2023). Monomers used during polymerization typically already contain mono- or disaccharides. However, labile moieties including sialic acid or sulfate containing glycans can be attached post-polymerization using orthogonal chemistries (Kramer et al., 2015; Mastrotto et al., 2022; Agard et al., 2004).

Polymer stereochemistry is a critical determinant of mucin mimetic function. Using stereo-controlled ROMP, Kruger *et al* recently demonstrated that *cis*-poly (norbornene) glycopolymers adopt extended conformations and could inhibit cholera toxin binding to GM1 ganglioside more effectively than *trans*-analogs, which adopt globular aggregates (Kruger et al., 2021). Using synthesized mucin polymers, stereochemistry-driven interactions of mucin mimetics with plant lectins, cell surface receptors, and bacterial toxins have been reported (Ishaq et al., 2025; Zhang et al., 2024; Delaveris et al., 2020; Werlang et al., 2021). Together, these studies establish stereochemistry as an essential design parameter in glycomaterial development. Most recently, Feldhof *et al* introduced double-brushed glycopolymers whose architecture mimics the natural bottle-brush architecture of mucins (Feldhof et al., 2025), opening an avenue to investigate the influence of higher-order architecture on structure-function relationship of mucins.

Limitations of mucin mimetic approaches include the non-native backbone chemistry, which may alter glycan conformational dynamics and preclude interactions dependent on the mucin polypeptide sequence. The triazole linkages generated by click chemistry, while stable, are conformationally rigid and can bias glycan orientation, with potential consequences for lectin recognition geometry and metabolic stability. Polymer-glycan conjugates may also exhibit different pharmacokinetic properties relative to native glycoproteins, including altered biodistribution and immunogenicity profiles. Outstanding questions include determining when the native mucin backbone is essential versus dispensable for biological function. Development of biodegradable scaffolds with tunable degradation kinetics, exemplified by recent thio-mucin and mirror-image mucin variants (Kohout et al., 2023), would enhance therapeutic applicability. Hybrid chemoenzymatic approaches that integrate enzymatically synthesized native O-glycans onto synthetic polypeptide (Wood et al., 2025; Delaveris et al., 2020) or carbon-based polymer scaffolds combine the structural authenticity of enzymatic synthesis with the tunability of mimetic platforms, and represent a promising convergence with other synthesis methods (Figure 2d).

## 6 Discussion

The three glycoengineering strategies occupy complementary positions in the experimental toolkit, each offering distinct trade-offs between structural control, scalability, and biological fidelity as summarized in Table 2.

**TABLE 2:** Comparison of glycoengineering approaches.

Parameter	Cellular glycoengineering	Cell-free synthesis	Mucin mimetics
Structural control	Core structure, site occupancy	Atomic-level precision	Valency, density, spacing
Homogeneity	Moderate (simplified)	High	High
Scalability	High (mg–g)	Low (μg–mg)	Moderate (mg)
Backbone	Native protein	Native peptide	Synthetic polymer
Primary applications	Cell-based assays, physical biology of mucins	Structural biology, microarray construction	Multivalency studies of glycan moiety
Validation methods	Sequencing of the engineered cells, lectin profiling using flow cytometry, glycomics and glycoproteomics	NMR, MS, HPLC, and lectin staining	SEC-MALS, NMR, and lectin staining
Key limitation	Residual heterogeneity, incorporation of non-natural sugar	Scalability, donor sugars are costly	Non-native backbone

### 6.1 Selection criteria

Cellular glycoengineering is optimal when native protein folding, trafficking, and membrane presentation are essential, for instance, in cell-based binding assays, studies requiring full-length mucins with authentic domain architecture, or investigations of glycan function in cellular context. Chemoenzymatic synthesis is preferred when atomic-level structural definition is paramount, such as for biophysical characterization by NMR or crystallography, structural biology of glycan-protein complexes, or construction of glycan microarrays with defined specificities. Mucin mimetics are most appropriate for systematic dissection of multivalency effects, where independent variation of valency, density, and spacing is required to establish structure-activity relationships or when large quantities of material are needed.

### 6.2 Convergent strategies and hybrid workflows

The boundaries between cellular, chemoenzymatic, and mimetic platforms are increasingly permeable. In the cellular-to-chemical workflows, glycoengineered cells produce mucin scaffolds bearing simplified, homogeneous O-glycans in high yield, and are then enzymatically remodeled or chemically conjugated to extend the accessible structural space (Nason et al., 2021; Jaroentomeechai et al., 2024; Jaroentomeechai et al., 2022). Cellular-with-cell-free format performs glycoprotein synthesis and glycan modification in either cell-based or cell-free platforms, selecting whichever is possible and more efficient for each step (Jaroentomeechai et al., 2022; Hamilton et al., 2017). Lastly, chemical-to-enzymatic workflow elaborates synthetic polymer or polypeptide scaffolds by chemoenzymatic glycan installation to combine mimetic tunability with native O-glycan authenticity (Figure 2d). Lipid-anchored chemoenzymatic mucin analogs that retain native α-GalNAc–Ser/Thr linkages while permitting systematic variation of glycan capping exemplify this paradigm and enable dissection of mucin–binder interactions directly at the cell surface (Wood et al., 2025; Delaveris et al., 2020). Each paradigm targets a distinct limitation of single-platform approaches including scalability, glycoenzyme accessibility, or backbone authenticity.

Metabolic incorporation of non-natural monosaccharides bearing bioorthogonal handles (azides, alkynes, ketones, cyclopropenes) further bridges cellular and chemical approaches, enabling site-selective conjugation of reporter groups or therapeutic payloads to cellular glycoproteins (Wang and Mooney, 2020). Despite these advances, scalability remains a critical bottleneck. Chemoenzymatic synthesis, despite its precision, currently operates at scales insufficient for clinical development. Nucleotide-sugar donors represent a substantial cost driver but enzymatic regeneration systems utilizing inexpensive starting materials offer partial mitigation but add process complexity (Hanson et al., 2004; Yu and Chen, 2016). Cellular glycoengineering in mammalian hosts faces inherent limitations in volumetric productivity and cost as well as increasing glycoform heterogeneity for a more complex glycan structure. Prokaryotic platforms and cell-free systems may offer scalability advantages, though demonstration of gram-scale production of defined mucin glycoforms with therapeutic-grade purity remains an outstanding challenge for the field.

### 6.3 Emerging technologies for O-glycan engineering

Beyond the aforementioned, several technologies are positioned to address bottlenecks in mucin O-glycan synthesis. Engineered *Saccharomyces cerevisiae* strains with rewired UDP-sugar metabolism now provide preparative access to UDP-GlcA, UDP-Xyl, UDP-Fuc, and rare UDP-pentoses required for natural product glycosylation (Crowe et al., 2024), while metabolically engineered *E. coli* and *Bacillus subtilis* can deliver Neu5Ac at titers approaching industrial scale, addressing one of the cost drivers in chemoenzymatic sialylation (Dang et al., 2025). Continuous directed evolution platforms such as yeast’s orthogonal DNA replication system (OrthoRep) or analogous system in *E. coli* (BacORep) have emerged as a route to expand or alter enzyme substrate specificity or improve catalytic activity (Rix et al., 2020; Tian et al., 2023), and are readily adaptable for GT evolution. Rational design such as bump-and-hole engineering of ppGalNAcT provides a protein-engineering strategy in which GalNAc-T isoforms are mutated to accept bioorthogonal UDP-GalNAc analogs. The GalNAc-T mutant enables isoform-specific tracking of O-glycosylation sites in living cells. This approach could potentially be used to resolve the substrate repertoires of individual GalNAc-Ts within the family (Schumann et al., 2020).

These advancements are increasingly enabled by parallel progress in computational and analytical infrastructure. Deep learning-based structural predictors such as AlphaFold3 can now accommodate glycan-protein complexes (Abramson et al., 2024). Huang *et al* recently provided a critical assessment and validated approach for modeling stereochemically accurate glycan-protein interaction using bondedAtomPairs (BAP) syntax as input for AlphaFold3 prediction (Huang C. et al., 2025). Although current predictions of glycan-bound complexes still depend on user expertise to specify glycosidic linkages and stereochemistry (Huang C. et al., 2025), integration with mucin O-glycan structural databases and glycoengineering datasets may enable predictive design of mucin-binder interactions in the near term. On the analytical side, single-cell glycomics analysis using DNA-barcoded lectin probes (scGR-seq/SUGAR-seq) (Minoshima et al., 2021; Kearney et al., 2021) or lectin profiling by cytometry by time-of-flight (CyTOF-Lec) have been reported (Ma T. et al., 2022). The single cell analysis can help screen or validate glycoengineered cell lines used as production hosts. Together, these emerging synthetic biology, computational, and analytical tools are expected to alleviate current constraints in mucin O-glycan engineering and production.

### 6.4 Toward mucins from other species

Current glycoengineering strategies have focused predominantly on human mucins, however, expanding these approaches to non-human organisms may offer distinct opportunities, both in dissecting biology governed by mucins and in biotechnological applications. Mucins are evolutionarily conserved from hydra to humans (Lang et al., 2016), and their O-glycosylation exhibits substantial interspecies variation in core structure utilization, sialylation patterns (including Neu5Gc and Kdn distribution), and terminal modifications, and these differences can have functional consequences for host-microbe interactions (Varki, 2010). The aforementioned approaches can, in principle, enable cross-species mucin production by: (a) heterologous expression of non-human mucin sequences in glycoengineered mammalian cell lines, which permits production of species specific mucin with defined O-glycoforms; (b) exploitation of alternative expression hosts such as insect cells, which produce mucin-type O-glycans with non-human modifications including hexuronic acid and phosphocholine substitutions (Gaunitz et al., 2013); (c) chemoenzymatic remodeling of native animal mucins using defined glycosyltransferase and glycosyl hydrolase panels, preserving native site occupancy pattern while installing specific terminal modifications; and (d) total chemical synthesis of O-glycomucins once the natural mucin and its O-glycans have been characterized. These approaches facilitate comparative studies of lectin-glycan recognition across species, development of structurally defined glycan-based prebiotics, and investigation of microbial adhesin specificity relevant to anti-infective strategies.

## 7 Conclusion

Cellular glycoengineering, chemoenzymatic synthesis, and mucin mimetics provide complementary access to structurally defined mucins and their O-glycans. As hybrid workflows become routine and analytical, computational, and synthetic infrastructure converge, the field is poised to deliver mucin glycoconjugates with more complex and biologically relevant O-glycan structures with better fidelity and at scale. The decision framework, matching production method to experimental objective, outlined here should help researchers navigate the mucin research landscape and identify the platform best suited to their specific structure–function questions.
